# Poisoning by Burning Gas

**Published:** 1878-01

**Authors:** 


### POISONING BY BURNING GAS.

The *Lancet* urges the inconvenience, and even danger, of the ordinary burning gas, it says :—

"To have not rooms pleasantly illuminated with gas is to undergo a process of poisoning, the more disastrous because, instead of directly producing the characteristic symptoms of defective blood oxygenation, the gas-polluted atmosphere insidiously lower the tone of vitality, and establishes a condition favorable to disease. It would be difficult to overrate the importance of this househood peril. Pictures are spoiled by gas, gilt mouldings are tarnished, the colors of decorated walls and ceilings fade, and men and women of delicate organization are enfeebled and injured by the foul air in which gas is discharged and supposed to burn innocuously. The extent to which this evil works in the midst of domesticated families during the long evenings is not adequately appreciated. After the first few unpleasant experiences are over, the physical sensibility becomes inured to the immediate results of breathing an atmosphere charged, more or less heavily, with the products of combustion and unconsumed coal gas. It is not creditable to the ingenuity of practical men that no method has yet been devised by which the advantages of gas as an illuminating agent may be secured without the drawback of slow poisoning, with the host of maladies a depressed vitality is sure to bring in its train."

